# Bacterial sensitivity distributions for biocides and metals

**DOI:** 10.1093/femsec/fiag075

**Published:** 2026-07-10

**Authors:** Daniel Jaen-Luchoro, D G Joakim Larsson, Johan Bengtsson-Palme

**Affiliations:** Department of Clinical Microbiology, Sahlgrenska University Hospital, 413 46 Gothenburg, Region Västra Götaland, Sweden; Centre for Antibiotic Resistance Research in Gothenburg (CARe), University of Gothenburg, 405 30 Gothenburg, Sweden; Division of Systems and Synthetic Biology, Department of Life Sciences, SciLifeLab, Chalmers University of Technology, 412 96 Gothenburg, Sweden; Centre for Antibiotic Resistance Research in Gothenburg (CARe), University of Gothenburg, 405 30 Gothenburg, Sweden; Department of Infectious Diseases, Institute for Biomedicine, University of Gothenburg, 405 30 Gothenburg, Sweden; Centre for Antibiotic Resistance Research in Gothenburg (CARe), University of Gothenburg, 405 30 Gothenburg, Sweden; Division of Systems and Synthetic Biology, Department of Life Sciences, SciLifeLab, Chalmers University of Technology, 412 96 Gothenburg, Sweden; Department of Infectious Diseases, Institute for Biomedicine, University of Gothenburg, 405 30 Gothenburg, Sweden

**Keywords:** biocide, metal, antimicrobial resistance, MIC, co-selection, co-resistance

## Abstract

Understanding how potent metals and biocides are for inhibiting bacterial growth is key for understanding risks for ecological disturbances and for co-selection of resistance to antibiotics. The aim of this study was to gather MIC data from the public literature to generate a centralized biocide and metal minimum inhibitory concentration (MIC) dataset, emulating established specialized datasets for antibiotics. The resulting dataset contains data for 53 antibacterial biocides, 21 metals, and 17 related compounds, collected from 289 publications between 1973 and 2024. The dataset includes 20 378 MIC values across 164 bacterial species, with a strong overrepresentation of clinically relevant organisms such as *Staphylococcus aureus* and *Escherichia coli*. Biocides like chlorhexidine and benzalkonium chloride, as well as metals such as copper, zinc, arsenic, cadmium, and silver, were the most represented compounds in each case. Some inconsistent MIC values were observed, complicating cross-study comparisons. The data collected in this study emphasizes the urgent need for standardized susceptibility testing methodology and consistent terminology for research on reduced susceptibility to biocides and metals. By centralizing MIC data, this work provides part of the foundation for future efforts to assess ecological risks and co-selection with antibiotic resistance, identify data gaps, and support future regulatory evaluations.

## Teaser

We compiled over 20 000 MIC values for biocides and metals into a single dataset to enable better comparisons, reveal gaps, and could help guiding future resistance research and regulation.

## Introduction

Antibacterial agents include both organic biocides and certain metals with antimicrobial properties. They are used to kill or control bacterial growth, most often in a broad, relatively non-selective manner. Antibacterial biocides and metals are widely employed as antiseptics, disinfectants and additives in healthcare settings, agriculture, animal farming, food production, household cleaning, and personal care products, to name a few applications (McDonnell and Russell 1999, Russell 2003, Maillard 2005, Mendiguchía et al. 2006, Castillo et al. 2008, Burridge et al. 2010, Nachman et al. 2013). They are distinguished from antibiotics in that they are not used as medicines, except a few that are approved for external use (e.g. skin disinfectants). There are also settings where such compounds are used for entirely different purposes than controlling bacterial growth, but of course, regardless of the intention, they still display antibacterial properties. Due to their often high efficacy in limiting bacterial growth, antibacterial biocides and metals are of great importance, especially from a public health perspective. Their ubiquity and diverse applications increase the likelihood that biocides will enter the environment through various pathways (SCENIHR 2009), where bacteria in both human-managed settings and natural environments become exposed (Jones and Joshi 2021).

Exposure to biocides and metals in the environment, even at relatively low concentrations, may exert selective pressure on bacteria, potentially leading to reduced susceptibility (Knapp et al. 2013, Maillard et al. 2013, Oggioni et al. 2013, Wesgate et al. 2016), which may diminish the effectiveness of the products. While reported reduced susceptibility often does not reach concentrations typical of in-use levels (Nikaido 2003), the presence and persistence of pathogenic bacteria on previously decontaminated surfaces in healthcare facilities have been observed numerous times (Muto et al. 2003, Bhalla et al. 2004, Hota 2004, Kramer et al. 2006, Vonberg et al. 2008, Lawley et al. 2010, Otter et al. 2011). Additionally, the presence of biocides and metals in the environment can alter microbial community composition by reducing susceptible species and strains while favoring those with reduced susceptibility or degradation capabilities (Campa et al. 2019, Reiss et al. 2024). Such shifts may disrupt key ecological functions, including nutrient cycling, soil structure maintenance, and microbial primary production (Campa et al. 2019). This highlights the need for surveillance programs for monitoring the biocide landscape and proper data to asses such impacts (SCENIHR 2009).

The potential link between antibiotic resistance and reduced susceptibility to biocides/metals generates concerns about the possibility of promoting antibiotic resistance following exposure to these chemicals (Maillard 2007, SCENIHR 2009, Maillard and Denyer 2009, Maillard et al. 2013). Many genes providing reduced susceptibility towards biocides and metals have been described. These genes are sometimes located on mobile genetic elements, such as plasmids, alongside antibiotic resistance genes, raising the risk of co-selection (Pal et al. 2015). Another mechanism behind co-selection is the cross-resistance between biocides and antibiotics, also raised by the European Comission and the Scientific Committee on Emerging and Newly Identified Health Risks (SCENIHR 2009, 2010). Here, reduced susceptibility is provided by a single, common mechanisms effective against both biocides and antibiotics, and often includes efflux pumps, modifications at the cell wall level or metabolic adaptations (Tattawasart et al. 2000a,b, Piddock 2006, Webber et al. 2008, Maillard et al. 2013, Grande Burgos et al. 2016).

Defining resistance to biocides and metals is more complex than for antibiotics. Clinical resistance to antibiotics in pathogens is based on treatment efficacy in relation to therapeutic concentrations. To assess risks for therapy failure, breakpoints based on minimum inhibitory concentration (MIC) are set following standardized guidelines as defined by the European Committee on Antimicrobial Susceptibility Testing for Europe (EUCAST 2025) or the Clinical and Laboratory Standards Institute for the United States (US) (CLSI 2024). Bacteria that grow at, or above, these MICs are considered clinically resistant, as the antibiotic would likely no longer be effective at therapeutic concentrations. In contrast, biocides are not used for systemic treatment, making this definition inapplicable. Additionally, the lack of standardized methodologies makes MIC data for biocides and metals difficult to compare across studies. Another definition is the microbiological one, referring to bacterial strains with MICs higher than those of the wild-type population of the same species, without considering threapeutic efficacy. These can be identified using epidemiological cut-off values (ECOFFs) as reported by EUCAST, which distinguish between wild-type populations and those with acquired resistance. While extensive MIC data allow the establishment of ECOFFs for antibiotics (EUCAST 2024), such comprehensive and documented data are lacking for biocides, making difficult to apply a similar concept. In the absence of standardized clinical breakpoints or well-defined wild-type distributions, we therefore use the term reduced susceptibility here in an operational, phenotypic sense to denote bacterial isolates or subpopulations exhibiting elevated MICs relative to a reference or baseline within a species. In particular, reduced susceptibility is used to describe shifts in MIC distributions, including the presence of subpopulations with clearly higher MICs than the main population, without implying specific thresholds or clinical relevance.

This study represents, to the best of our knowledge, the first attempt to compile and present large-scale MIC data for antibacterial biocides and metals. By compiling such data across species, methodologies, and studies, we aim to provide a resource that potentially could become useful in risk assessment, both for ecotoxicological effects on bacterial communities, but also for initial assessments of risks for co-selection of antibiotic resistance. In addition, we also believe such a compilation of MIC data will enhance understanding of reduced susceptibility to biocides and metals and better highlight data inconsistencies and knowledge gaps across the field. Thereby, this work serves as a stepping stone toward future standardization efforts.

## Material and methods

### Data collection

An extensive literature search was conducted in PubMed (https://pubmed.ncbi.nlm.nih.gov/), using different combinations of terminology related to biocides, metals, MIC, and tolerance/resistance (Supplementary File S1). The literature search was conducted between September 2023 and March 2024. No year restrictions were established, and no limitations were set for specific biocides or metals. Only papers written in English were considered. The list of papers obtained with the different combinations of terminology were merged, and duplicates were removed.

Each article was manually reviewed following the steps sumarized in Supplementary Fig. S1. The MIC data, stated as the lowest concentration preventing visible cell growth, were recorded, along with methodology, culture media, bacterial species and strain, source of isolation, geographical location, and the digital object identifier (DOI) of the original study. No restrictions were set regarding the media used, incubation time, temperature, or methodology. Data not clearly linked to a specific bacterial species or related to nanoparticles were excluded. Additionally, only MIC values measured in micrograms per milliliter (µg/ml), or those convertible to this unit, were included to facilitate data comparison. MIC values were collected as reported in the original publications and if necessary converted to µg/ml. For metal MICs expresed in units other than µg/ml, the metal ion molar mass was used for unit conversion (i.e. mM, µM). Based on molecular masses of ions and common salts used, the maximum anticipated error introduced was still minor with very limited impact expected on the overall MIC distributions.

### Data classification

Collected data were organized into tables and characterized by the number of data points, families, genera, and species for each biocide and metal using in-house scripts. Species-level data were log₂-transformed and visualized in heatmaps using Python’s Pandas, Seaborn, and Matplotlib libraries.

### MIC distributions

MIC values for bacterial species were grouped into predefined bins (0.001 µg/ml to >1000 µg/ml) and categorized using Pandas. Plots were generated with Matplotlib, with species arranged alphabetically for consistency.

## Results and discussion

### Summary of collected data

**Figure 1:**
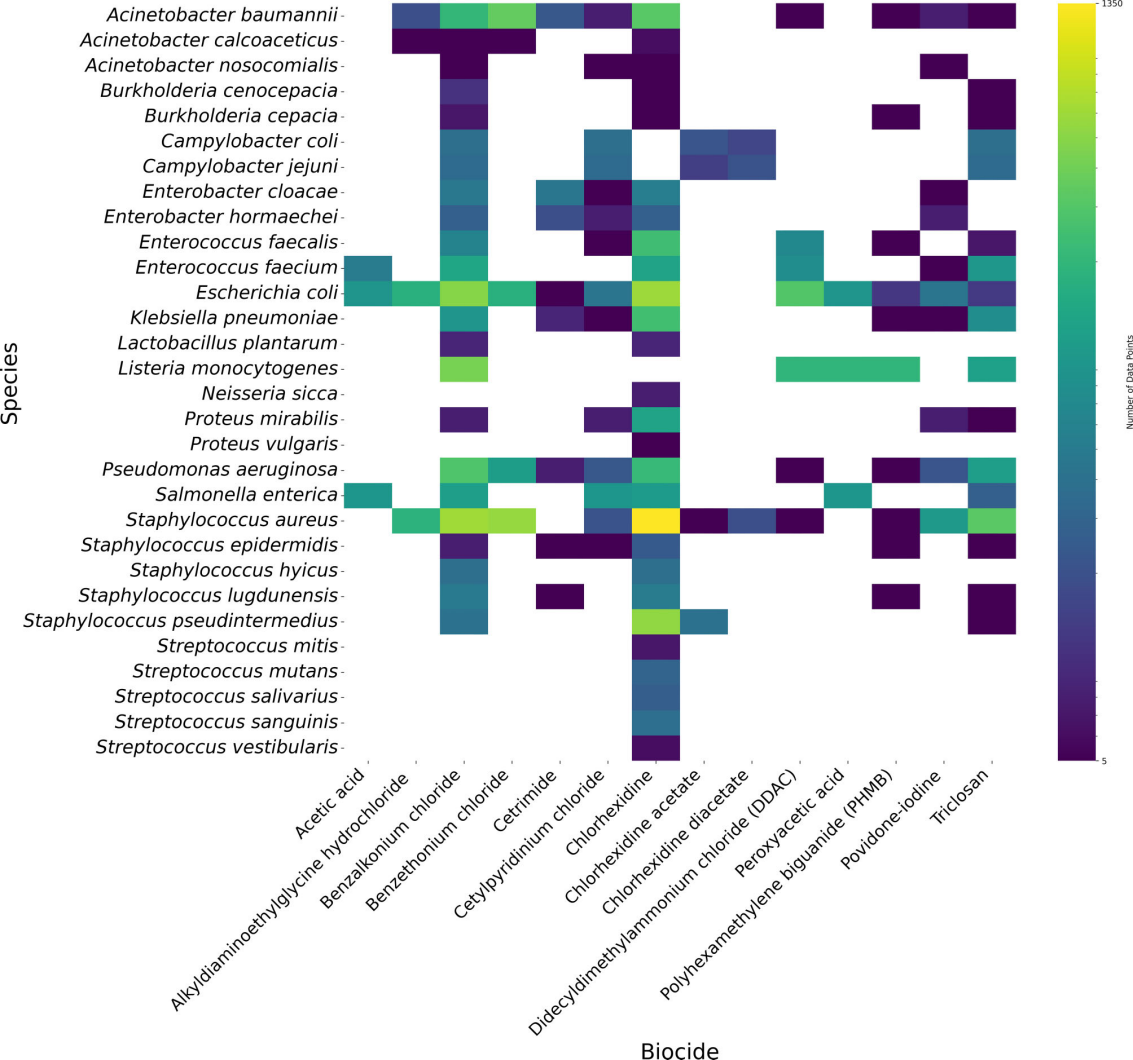
Heatmap of the distribution of MIC data for each biocide across different species. Only species and biocides with at least five MIC datapoints were used. The data ploted gathers 30 species, 14 biocides (representing 26.4% of all biocides), and 11 647 datapoints, accounting for 73.6% of the total biocide MIC data collected.

**Figure 2:**
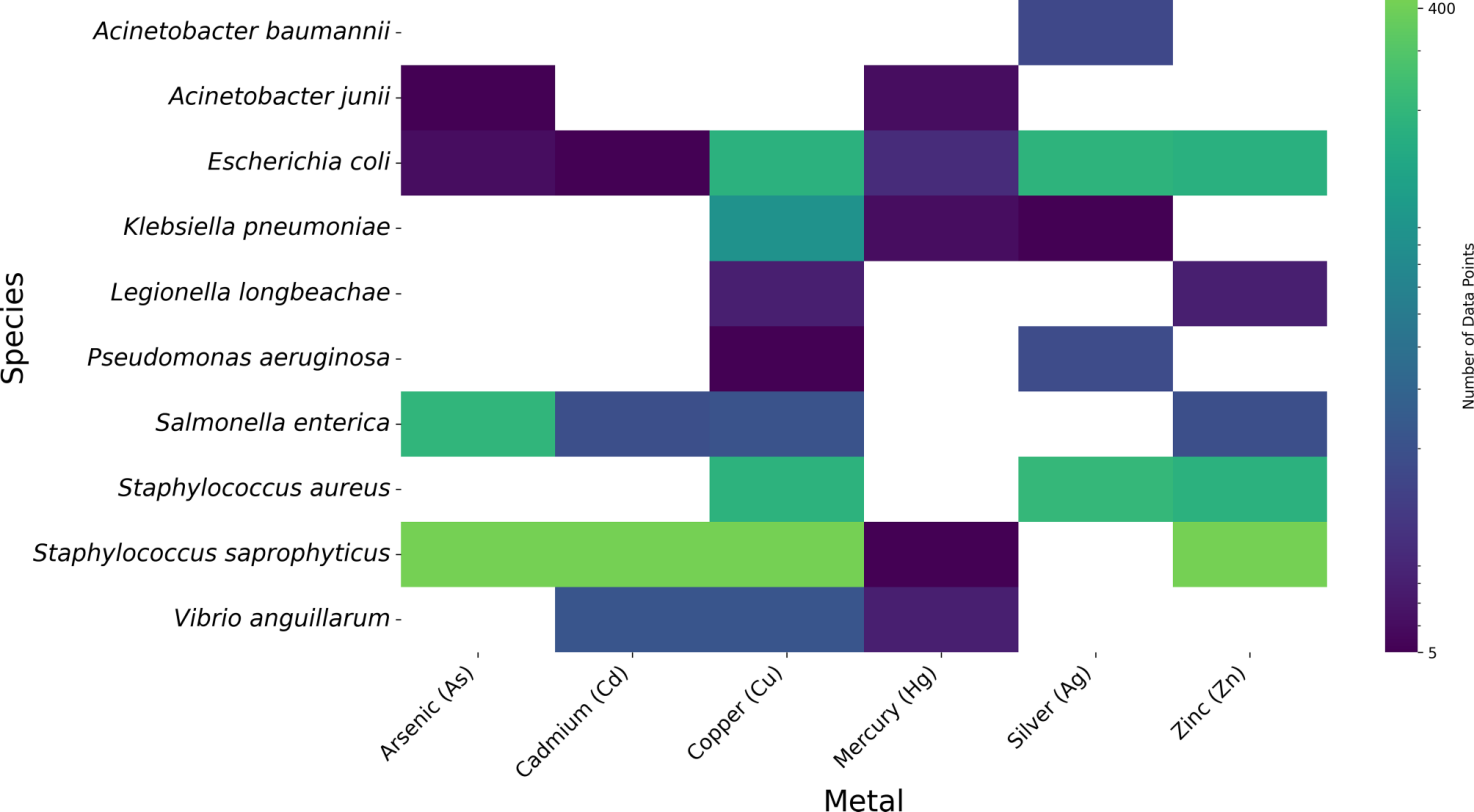
Heatmap of the distribution of MIC data for each metal across different species. Only species and metals with at least five MIC datapoints were used. The data represented corresponds to 10 species, 6 metals (28.5% of all metals), and 3285 datapoints, representing 94.2% of the total metal data collected.

Our initial literature search yielded a total of 2003 papers. After the first manual curation, 289 papers published between 1973 and 2024 containing data listed as “bacterial MIC” meeting the desired criteria, were included in the present study. Of these, 130 papers provided MIC data for biocides, 168 for metals, and 19 for other compounds not classified as either biocides or metals, but used for similar antibacterial purposes. After additionaly filtering out papers reporting MIC data for metal-nanoparticles, only 34 papers included suitable data on metals. Some papers included MIC data for several types of compounds. The total number of MIC datapoints collected was 20 378, and these were classified according by specific biocide, metal or other compound (Supplementary Files S2, S3, and S4). Data is summarized in Tables 1–3, and corresponded to 53 biocides, 21 metals and 17 additional compounds. Distributions of the number of datapoints per species for biocides and metal are visualised in Figs. 1 and 2, respectively, showing only data for bacterial species and biocides/metals with at least five datapoints.

**Table 1:** Number MIC data points per biocide, along with the minimum, maximum, and median MIC values (µg/ml), and the number of bacterial families, genera, and species represented in each case.

Biocide	MIC count	Min MIC (µg/ml)	Max MIC (µg/ml)	Median MIC (µg/ml)	Family count	Genus count	Species count
1,3 dibromo, 5,5-dimethylhydantoin	95	312	3648	1824	1	1	1
1,3-Propanediamine-N-(3-aminopropyl)N-dodecyl	4	1.25	5	2.5	1	1	1
Acetic acid	238	820	3280	2048	2	3	3
Acidified sodium chlorite	94	80	1280	320	1	1	1
Acriflavine	356	2	64	8	2	3	3
Alkyldiaminoethylglycine hydrochloride	386	0.06	800	0.125	3	3	7
Benzalkonium bromide	158	0.63	64	32	3	3	3
Benzalkonium chloride	2833	0.016	1000	8	20	28	69
Benzethonium chloride	1203	0.25	800	12.5	4	4	8
Benzisothiozolinone	9	1	125	15.6	5	7	8
Camphorated paramonoclorophenol	8	85	680	340	4	4	4
Carvacrol	3	662	1200	1200	3	3	3
Cetrimide	120	0.4	232	32	7	9	17
Cetrimonium bromide (CTAB)	3	1	16	8	2	3	3
Cetylpyridinium chloride	331	0.12	5120	8	18	28	43
Chlorhexidine	4711	0.063	4000	2	35	55	118
Chlorocresol (p-chloro-m-cresol)	93	500	1000	500	1	1	1
Clorine	165	250	250	250	1	1	1
Crystal violet	1	32	32	32	1	1	1
Dequalinium chloride	1	1	1	1	1	1	1
Dibromopropamidine isethionate	6	50	75	75	1	1	1
Didecyldimethylammonium chloride (DDAC)	646	0.05	122	1.25	7	9	12
Dodecyltrimethylammonium chloride	92	128	512	256	1	1	1
Ethidium bromide	115	0.5	512	1	2	2	4
Eugenol	16	4	613	4	1	1	1
Formaldehyde	143	40	160	80	2	2	2
Hexachlorophene	70	64	128	128	1	1	5
Hexadecylpyridinium chloride	68	2	256	64	1	2	6
Hexadecylpyridinium chloride monohydrate (HDPCM)	78	1	32	4	1	1	1
Hexadecyltrimethylammonium bromide	94	40	160	80	1	1	1
Hexadecyltrimethylammonium chloride	95	40	160	80	1	1	1
Hexamidine	33	2	256	16	7	13	19
Hydrogen peroxide	293	4	256	125	4	4	4
Isothiocyanate	4	225	500	362.5	4	4	4
Limonene	2	20	40	30	2	2	2
Methylene blue	1	93.75	93.75	93.75	1	1	1
N-(3-aminopropyl)-Ndodecylpropane-1 3-diamine	197	2	16	4	1	1	1
Octenidine	12	1.95	125	3.9	3	3	3
Paracetic acid	37	156	625	313	1	1	1
Paramonochlorophenol	9	93.33	213.33	194.17	6	6	9
Peroxyacetic acid	385	156	1760	500	2	3	3
Phenol	12	750	1500	1125	8	9	10
Polyhexamethylene biguanide (PHMB)	236	0.2	58	3.1	10	13	18
Polyhexamethylene guanidine hydrochloride	12	0.24	125	7.81	6	7	9
Polyhexanide	25	0.25	1	0.5	1	1	1
Povidone-iodine	227	7.82	10 000	4375	10	12	24
Propanol-based mecetronium ethyl sulphate	98	664	42 500	1328	1	1	1
Sodium hypochlorite	331	312.5	50 000	1250	7	8	12
Thymol	17	0.5	3000	600	7	10	12
Triclocarban	19	0.08	0.125	0.08	1	1	1
Triclosan	897	1e-05	512	0.25	18	23	39
o-phenylphenol (OPP)	367	125	4000	1000	2	2	2
p-chloro-m-xylenol (PCMX)	375	12.5	4000	125	6	6	9

**Table 2:** Number of MIC data points per metal, along with the minimum, maximum, and median MIC values (µg/ml), and the number of bacterial families, genera, and species represented in each case.

Metal	MIC count	Min MIC (µg/ml)	Max MIC (µg/ml)	Median MIC (µg/ml)	Family count	Genus count	Species count
Aluminium (Al)	1	60 357	60 357	60 357	1	1	1
Antimony (Sb)	1	889	889	889	1	1	1
Arsenic (As)	633	6	6656	400	3	6	8
Barium (Ba)	1	122 130	122 130	122 130	1	1	1
Cadmium (Cd)	476	0.1	8192	50	6	9	12
Calcium (Ca)	8	781.25	6250	1171.875	4	4	4
Chrome (Cr)	10	77	900	278	4	5	6
Cobalt (Co)	22	0.1	567	4	2	3	4
Copper (Cu)	938	0.05	16 000	1600	9	12	19
Gallium (Ga)	2	12.8	12.8	12.8	1	1	1
Iron (Fe)	6	97	4222	4000	3	4	4
Lead (Pb)	49	0.2	2586	100	4	6	7
Manganese (Mn)	7	0.97	1965	15.6	2	2	5
Mercury (Hg)	43	1	73.9	4	7	11	15
Molybdenum (Mo)	1	241 950	241 950	241 950	1	1	1
Nickel (Ni)	10	7.8	463	135.865	4	4	8
Silver (Ag)	457	0.025	160	3.4	13	17	23
Sodium (Na)	1	228.3	228.3	228.3	1	1	1
Strontium (Sr)	1	79 265	79 265	79 265	1	1	1
Tin (Sn)	1	3524	3524	3524	1	1	1
Zinc (Zn)	818	0.05	3200	512	5	7	13

**Table 3:** Number of MIC data points per other compound, along with the minimum, maximum, and median MIC values (mg/ml), and the number of bacterial families, genera, and species represented in each case.

Other antibacterial compounds	MIC count	Min MIC (µg/ml)	Max MIC (µg/ml)	Median MIC (µg/ml)	Family count	Genus count	Species count
Chitosan	12	8	450	67	5	5	7
Citric acid	95	1578	12624	3156	1	1	1
Ethanol (EtOH)	179	6.25	50	25	1	1	1
Formic acid	50	1024	1024	1024	1	1	1
Geraniol	3	2125	4250	4250	2	2	2
Glydant (DMDM hydantoin)	9	187.5	375	375	5	7	8
Hydroxiapatite	3	4	16.1	8	2	2	3
Lactic acid	95	3776	7552	7552	1	1	1
Luteolin	2	312.5	312.5	312.5	2	2	2
Miconazole	198	1	256	2	1	1	2
Potassium persulphate	4	2500	2500	2500	1	1	1
SDS	73	128	2048	512	1	1	2
Sodium fluoride (NaF)	2	600	625	612.5	1	1	1
Sodium metasilicate	95	30 160	120 640	60 320	1	1	1
Tetraphenylphosphonium bromide	72	4	128	8	1	1	2
Thymoquinone	8	25	50	37.5	3	3	5
Trisodium phosphate	168	2	37 712	37 712	2	2	3

Overall, biocide data was biased to particular compounds, largely corresponding to their use as antibacterial agents. The most represented biocides were chlorhexidine and benzalkonium chloride, followed by benzethonium chloride and triclosan (Table 1). Chlorhexidine, a biguanide listed on the World Health Organization’s (WHO) List of Essential Medicines (WHO 2021), is extensively used as a disinfectant and antiseptic. It is widely used in healthcare settings for the disinfection of skin, wounds, and hospital materials, and plays a significant role in dental care and surgery. Additionally, chlorhexidine is used as an additive in cosmetics and pharmaceutical products, and it is also used in veterinary settings (Güthner et al. 2006, Kolahi and Soolari 2006, Maddison et al. 2008, James et al. 2017). This highlights its importance and may explain why it is the biocide for which most MIC data has been determined. Second to chlorhexidine, benzalkonium chloride is a quarternary ammonium compound (QAC) that has been extensively used across domestic, agricultural, industrial and clinical settings. It is used in personal hygene and cosmetic products, surface and tool desinfection, in water systems and as a preservant (Merchel Piovesan Pereira and Tagkopoulos 2019). Even though benzethonium chloride (another QAC) and triclosan (phenolic compound) have similar applications as the two previously mentioned biocides, they are not as extensively used (Fuchsman et al. 2022), which may explain why less MIC data were available. Actually, rising concerns about effects of triclosan on health has lead to a decrease in its use, and the US Food and Drug Administration (FDA) agency has even banned its applications in some products (FDA 2016).

Regarding metals, copper and zinc had the most MIC data collected, followed by arsenic, cadmium and silver (Table 2). Metals such as silver and copper have been historically important to humanity since ancient times due to their antimicrobial and preservative properties (Gupta and Silver 1998, Lansdown , Sudha et al. 2009). Today, these two metals continue to be widely studied for their bactericidal properties, quite often in the form of nanoparticles and surface coatings, which have significant applications in water and air purification, wound dressings, and as therapeutic or disinfectant agents (Deshmukh et al. 2019, Salah et al. 2021, Ren et al. 2024). However, these metals are far more widely used for purposes other than their antibacterial properties.

Regarding other compounds not classified neither as biocides nor as metals, those with the most data collected included miconazole, ethanol, and trisodium phosphate (Table 3). However, the differences in the number of data points are not as pronounced as for the two previous categories.

### Sensitivity data collected is biased towards clinically relevant bacteria

The dataset collected in the present work contained MIC data for 164 different bacterial species, corresponding to 71 genera and 43 families. The vast majority of species were Gram negative (Supplementary Fig. S2). Regarding number of species per family, most families included between 1 to 5 species (Supplementary Fig. S3). However, some families, generally those more related to clinical settings, were overrepresented, with the top one family being the *Enterobacteriaceae*, with 19 species (Supplementary Fig. S3). Nevertheless, it is also important to consider the source of isolation to better understand the structure of the dataset. Of the total dataset, 27.5% originate from human or clinical sources, while 29.5% correspond to isolates associated with, e.g. animal farming, food production, wastewater, industrial activities, as well as other environmental sources. Unfortunately, 42.8% of the data could not be clearly linked to a specific source, making it difficult to assess their contribution to the overall dataset structure. This highlights the importance of clearly reporting the isolation source to facilitate data interpretation and achieve a deeper understanding of the impact of biocides and metals in both environmental and clinical settings, as well as the relationships between these two contexts.

Filtering the data by species, only seven species accounted for 77.6% of the total MIC dataset (Table 4). Notably, the two species with the largest number of MIC data points—*Staphylococcus aureus* and *Escherichia coli*—alone represented 39.7% of all the data collected (Table 4), which is a significant contrast in comparison to other species. This congregation of data on a few species reflects their importance to human and animal health, as these pathogens or opportunistic pathogens are frequently responsible for infections and outbreaks. At the same time, *E. coli* is an important indicator of fecal contamination and widely used for assesing water quality (Odonkor et al. 2013), and *S. aureus* has also been proposed as a potential quality indicator for seawater and beaches (Topić et al. 2021, Steadmon et al. 2024), which makes these species relevant for monitoring environmental impacts of pollution.

**Table 4:** Top species with most data represented in the MIC dataset.

Species	Family	Biocides	Metals	Other compounds	Total
*Staphylococcus aureus*	*Staphylococcaceae*	4150	573	112	4835
*Escherichia coli*	*Enterobacteriaceae*	2676	578	6	3260
*Listeria monocytogenes*	*Listeriae*	1802	0	183	1985
*Salmonella enterica*	*Enterobacteriaceae*	1091	294	380	1765
*Staphylococcus saprophyticus*	*Staphylococcaceae*	1	1695	1	1697
*Acinetobacter baumannii*	*Moraxella*	1207	17	1	1225
*Pseudomonas aeruginosa*	*Pseudomonadaceae*	1016	21	2	1039

When analyzing the distribution of source data for these species, clear biases toward one context or the other are observed. For *S. aureus*, 31.4% of the data points are linked to human or clinical sources, while only about 10% originate from non-human sources, mainly associated with animals. In the case of *E. coli*, ∼38% of the data derive from human or clinical isolates and 30% from other sources, mostly related to animal farming and the food industry. In contrast, other species show strong biases toward specific contexts. For example, 84.8% of *Listeria monocytogenes* isolates are associated with animal farming and environmental sources, whereas 91.8% of *Salmonella enterica* data are linked to human or clinical settings.

Data covering a wider range of species and providing a clear indication of the source of origin would be valuable for assessing the potential impact on microbial communities and different ecological niches. Such informationwould allow a clearer differentiation between interconnected environments, improve our understanding of their interactions, and clarify how the use of biocides and metals may influence these systems, as well as the influence of the environment over the clinical context and viceversa.

In hospital settings, biocides are widely used to disinfect and decontaminate surfaces, tools, water systems, and other areas to eliminate potential pathogens. However, growing concerns about strains that survive these measures—particularly those with antibiotic resistance genes—have led to intensified research on clinically relevant pathogens and their reduced susceptibility or survival capacities (Muto et al. 2003, Bhalla et al. 2004, Hota 2004, Kramer et al. 2006, Vonberg et al. 2008, Lawley et al. 2010, Otter et al. 2011). Most studies, therefore, focus on isolates from hospitals and other care facilities, particularly on clinically relevant bacterial species such as the ones mentioned earlier, or model strains of the same species, to test the effects of these substances. Additionally, research often targets particular groups, such as carbapenem-resistant, methicillin-resistant *S. aureus* (MRSA) or extended-spectrum beta-lactamase (ESBL)–producing bacteria (Lambert 2004, Deus et al. 2017, Liu et al. 2017, Vijayakumar et al. 2018), since the research goal is often not only to evaluate reduced susceptibility to biocides or metals *per se*, but also what effect these compounds can have on co-selection of antibiotic resistance phenotypes. Co-selection due to use of biocides/metals might have influenced enrichment of antibiotic resistance bacteria, which would be especially worrisome in health-care facilities, veterinary environments or in food production (Maillard and Denyer 2009). Efforts should be made to expand the representation of relevant species and genera towards those found in the environment, particularly in environments where biocides and metals are frequently encountered at elevated concentraions. For example, several genera have been shown to be enriched, or to present marked reduced susceptibility, in metal contaminated areas, including *Acinetobacter, Marinobacter, Pseudomonas, Sulfobacilus, Halomonas*, and *Paracoccus* (Zhao et al. 2025). Some of these genera are completely absent in the colleted dataset. *Pseudomonas*, for example, is represented by 10 species, but 95% of the data corresponds to *Pseudomonas aeruginosa* and those are mostly derived from clinical or unknown sources. This makes difficult to assess the impact of this species at the environmental level. The remaining 5% (51 datapoints) corresponds to the other nine species, such as *Pseudomonas nitroreducens*, representing an insufficient amount of data for drawing conclusions.

By expanding MIC data towards environmental contexts, as well incorporating detailed metadata, would allow to better understand how relevant taxa and environmental factors influence susceptibility to biocides and metals. It is worth highlighting that susceptibility can be heavily influenced by surrounding conditions, and even small variations can lead to large differences. Laboratory settings should try reproducing environmental conditions as much as possible, both in terms of media composition and physicochemical parameters, in order to generate data that are more suitable for comparison with environmental conditions. Such data, together with a broader species range and detailed metadata, would substantially improve the ecological relevance and interpretability of these studies. At the same time, adapting experimental conditions to specific ecological settings might generate MIC values that are less comparable between studies, so the ecological relevance and comparability between studies need to be weighted against each other. Yet, such ecological knowledge is essential to identify the most critical focal points for intervention, as well as how these effects may translate into clinical settings.

### Collected data enables easy comparison of MIC distributions

**Figure 3:**
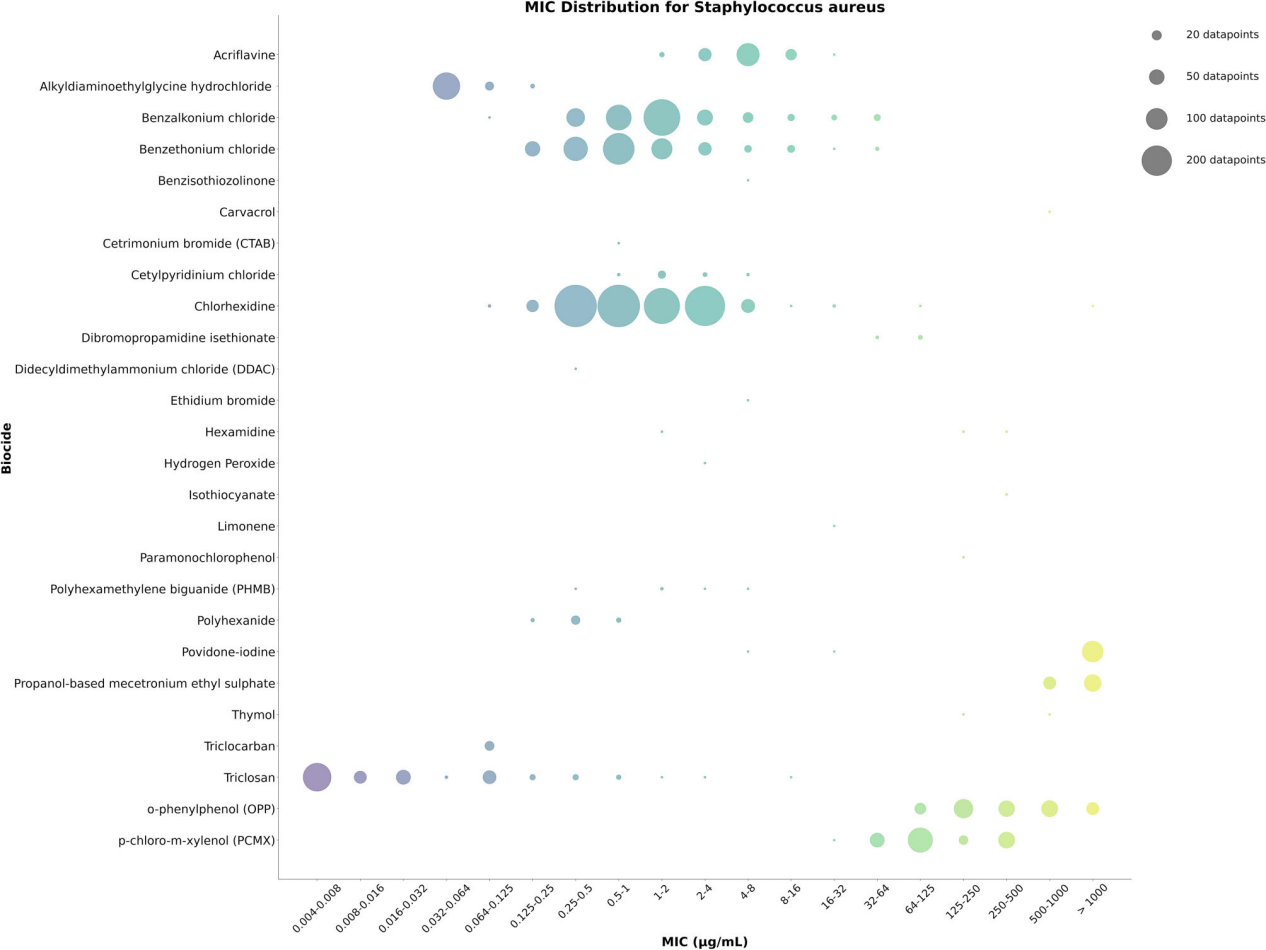
Biocides MIC distributions for *S. aureus*. A total of 4151 datapoints (26.2% of the total MIC data for biocides) representing 26 biocides are included.

Centralizing MIC data allowed us to study the MIC distributions for individual species. For example, the MIC distributions for biocides in *S. aureus* (Fig. 3, Supplementary File S5), the species with the highest number of datapoints in the present dataset, show similar distributions for compounds such as benzalkonium chloride, benzethonium chloride, and chlorhexidine, ranging mostly between 0.125 and 32 µg/ml. In contrast, *S. aureus* shows much higher MICs to compounds such as povidone iodine, propanol-based mecetronium ethyl sulfate, o-phenylphenol (OPP), and p-chloro-m-xylenol (PCMX), but is much more sensitive to triclosan compared to all the other biocides for which data have been collected (Fig. 3).

**Figure 4:**
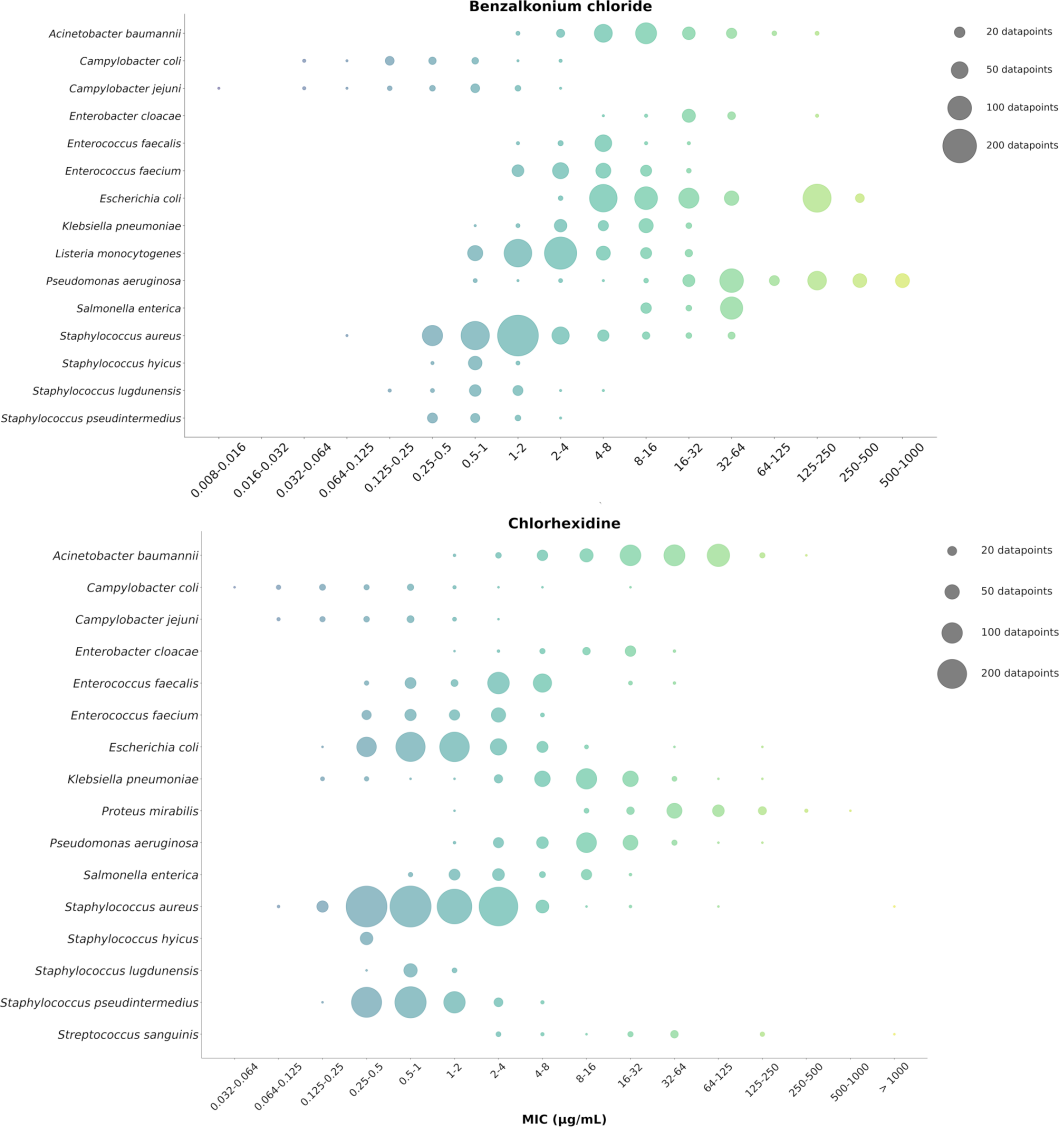
Benzalkonium chloride and chlorhexidine MIC distribution for species with at least 30 datapoints in the dataset. For benzalkonium chloride, a total of 15 species and 2671 datapoints are represented, corresponding to 21.7% of species and 96.6% of the data collected for this biocide. For chlorhexidine, 16 species (13.6%) and 4420 datapoints (93.8%) are represented from the total dataset for this biocide.

Distributions can also be studied and compared between different species. In Fig. 4, benzalkonium chloride and chlorhexidine MIC data for species with at least 30 datapoints is represented. Taking some of the mentioned clinically relevant species in Table 4, species such as *E. coli* and *A. baumannii* had similar MIC distributions (between 1 and 500 µg/ml) for benzalkonium chloride. In contrast, *S. aureus* had MICs ranging from 0.25 to 64 µg/ml, with most data points falling within the 1 to 2 µg/ml interval, and *P. aeruginosa* showing a shift towards higher concentrations (Fig. 4). For chlorhexidine, *E. coli* and *S. aureus* appeared to have similar distributions, with most data ranging from 0.125 to 16 µg/ml, with some data points at higher concentrations. Meanwhile, *A. baumannii* and *P. aeruginosa* showed a clear shift towards higher concentrations (i.e. less sensitivity to chlorhexidine). Other species such as *Campylobacter coli* and *Campylobacter jejuni* seemed to be generally more sensitive to both biocides compared to the other species represented in Fig. 4. Statistics associated to the data represented in Fig. 4 are found in Supplementary File S5.

Regarding metals, copper, zinc, arsenic and silver were the most represented metals in the collected dataset, as mentioned before. The distribution for these elements among the among species with at least 30 datapoints appeared to be quite similar for copper and silver in each case, while zinc showed variations in the range where the majority of data points were concentrated in each represented species. Silver was by far the most potent studied metal (notice different scales on the x-axes; Supplementary Fig. S4). An important limitation encountered when collecting MIC data for metals is the frequent lack of clarity regarding whether the reported MIC refers to the metal ion or to the metal salt. In many manuscripts, the nomenclature of the ion and the corresponding salt is used interchangeably, while others report only the salt without specifying whether the MIC value refers to the salt or to the metal ion itself. This lack of clarity significantly complicates data comparison, as the MIC for the same metal may differ depending on whether it is expressed in terms of the ion or the salt. Additional examples of MIC distributions for metals, as well as biocides and other species, are included in Supplementary File S6.

The MICs collected in the present work are reported as is, with only unit standardization to µg/ml performed whenever necessary, as stated in the methods section. Nevertheless, all data points are linked to the original publications (Supplementary Files 2, 3, and 4) to facilitate verification of the reported values. A recommended practice to overcome this issue would be to standardize the reporting of MIC data in terms of metal ions or salts, or at minimum to clearly specify the chemical form to which the MIC refers. This would substantially enhance comparability across different studies.

Overall, descriptive statistics (Supplementary File S5) indictates high variability in biocide and metal susceptibility across the different compounds and bacerial species represented. Biocides such as chlorhexidine, benzethonium chloride, and triclosan show lower MIC values, while other compounds such as phenolic or alcohol-based biocides generally require higher concentrations in comparison. When analyzing statistics for species distributions, Gram-positive bacteria are generally more susceptible than Gram-negative species. High variance values and broad MIC ranges for several compounds and species is indicative of a marked heterogeneity across studies and species.

Nevertheless, comparison and interpretation of the dataset collected is inherently limited by variability in the underlying methodologies, which will be discussed in the following section. These differences in experimental conditions may be responsible for some of the variability observed in the distributions and descriptive statistics, which is particularly relevant in the case of metals, as the bioavailability of these elements is strongly dependent on the media used. Also, some resistance phenotypes are only expressed during particular conditions, which can be the case for example with silver (Elkrewi et al. 2017) and copper (Hikal et al. 2024). For this reason, the distribution examples presented here should be interpreted with caution, and the collected metadata should be used to extract more comparable subdatasets based on similar MIC determination conditions.

### Comparisons across studies reveal inconsistencies in MIC data

One major challenge for collecting and integrating biocide and metal MIC data is the absence of standardized methodologies, as stated above. This issue has been raised previously (Maillard 2018), highlighting the need for consensus in this field, much like for clinical and veterinary antibiotic resistance. The method most widely used according to the collected data was broth microdilution (57%), followed by agar dilution (29%). Around 11% of the data was not possible to determine clearly which method was used for MIC determination, and the remaining 3% gathered data was associated to broth macrodilution, broth dilution, disk diffusion or the spot plate method. But even using the same method for a given species, variations in the cultivation conditions were evident. The lack of agreement often makes it difficult—or even impossible—to compare data across studies or to identify the most accurate MIC value. This challenge was particularly evident when measurements for a specific biocide or metal were taken for the same strain in different studies, but with different methodologies (Table 5).

**Table 5:** MIC values for chlorexidinde, measured for specific strains in different studies.

Species	strain	Biocide	MIC (µg/ml)	Assay	Media	PMID
*Staphylococcus epidermidis*	ATCC 12228	CHX	1	BMi	Tryptic soy broth	29126275
*Staphylococcus epidermidis*	ATCC 12228	CHX	2	AD	Mueller–Hinton agar	20705628
*Staphylococcus epidermidis*	ATCC 12228	CHX	16	BMi	Cation-adjusted Mueller–Hinton broth	30986480
*Streptococcus mutans*	ATCC 25175	CHX	0.7	BMi	Unknown	32173666
*Streptococcus mutans*	ATCC 25175	CHX	0.97	BMi	Unknown	36995882
*Streptococcus mutans*	ATCC 25175	CHX	3.3	BMi	Brain heart infusion broth	24818873
*Streptococcus mutans*	ATCC 25175	CHX	30	BMi	Tryptic soy broth	34941430
*Bacteroides fragilis*	ATCC 25285	CHX	2.44	BMa	Brucella broth	12238802
*Bacteroides fragilis*	ATCC 25285	CHX	9.75	BMi	RCM broth	12238802
*Staphylococcus aureus*	ATCC 25923	CHX	1	AD	Mueller–Hinton agar	33097426
*Staphylococcus aureus*	ATCC 25923	CHX	4	AD	Mueller–Hinton agar	20705628
*Staphylococcus aureus*	ATCC 25923	CHX	30	BMi	Tryptic soy broth	34941430
*Staphylococcus aureus*	ATCC 25923	CHX	32	BMi	Unknown	27916605
*Pseudomonas aeruginosa*	ATCC 27853	CHX	4	BMi	Mueller–Hinton broth	19456831
*Pseudomonas aeruginosa*	ATCC 27853	CHX	16	AD	Mueller–Hinton agar	20705628

BMi = broth microdilution; BMa = broth macrodilution; AD = agar dilution. CHX= chlorhexidine.

For example, for *S. aureus* ATCC 25923 (Table 5), the approach used for MIC determination—agar dilution (AD) and broth microdilution (BMi)—resulted in different MIC values. While each method yielded MICs that might appear similar or not significantly different within the same method, comparisons between AD and BMi measurements showed an order of magnitude difference. This discrepancy led to an MIC for chlorhexidine that was, on average, 12 times higher with respect to the BMi assay. This example might suggest that selecting solid or liquid media is the main cause, however also the media itself differed between these studies complicating the attribution of the MIC discrepancy to any one factor. In other cases, such as for *Streptococcus mutans* ATCC 25175, the same issue arised even when broth microdilution was consistently used. In that case, the primary difference was likely the type of media used; however, since not all studies specified the growth media it is again not possible to attribute differences to media alone.

Whether these changes in MIC values resulted from one or a combination of factors requires further investigation and is beyond the scope of this work. However, it is clear that unspecified or inadequately referenced parameters hinder reproducibility. In some studies, there is a significant lack of clarity in the experimental descriptions or no detailed information about the protocols and elements used. This can stem from missing details in the papers themselves or due to references that lead to inaccessible documentation. Overall, these gaps create additional challenges when comparing data or identifying factors that influence changes in MICs.

### Challenges in assessing susceptibility to biocides and metals

It has been suggested that studies on variations in susceptibility to biocides or metals should, in the long term, aim to assess the potential risk of reduced susceptibility associated with the use of a particular biocide, rather than merely reporting susceptibility profile variations (Maillard et al. 2013). Risk assessment frameworks are essential for companies and regulatory agencies, as they provide reliable information on the use of compounds and their potential to drive reduce susceptibility, akin to the approach used for antibiotics. The importance of this can be reflected, for instance, in the publication of the European Biocidal Products Regulation (BPR) (European Parliament, and Council of the European Union 2012) which requires manufacturers to ensure that the use of a specific compound does not lead to reduced susceptibility. Yet, the BPR, or other official regulatory bodies such as the US FDA (FDA 2016), do not indicate what protocols a manufacturer should follow in order to ensure and prove the safety of their compound (Maillard 2018). Although risk assessment is complex, it is feasible if reliable data—such as MIC values—are obtained using well-defined and reproducible methodologies (Maillard et al. 2013).

The MIC data collected in this study may contribute to building consensus by providing a clearer picture of MIC variability and helping to clarify which datasets can be meaningfully compared based on similar methodology used for its determination, and which ones need to be expanded. Comprehensive background MIC data, alongside additional key centralized datasets, such as minimum biocidal concentrations (MBC), a standardized methodology and risk assesment frameworks, are essential for advancing the study of reduced susceptibility to biocides and metals. Beyond this, the MIC data gathered here could be one of the pillars supporting regulatory actions, but several caveats exist, as the appropriate regulatory actions heavily depend on what risks are intended to be addressed; is the priority if the toxicity leads to impaired ecosystem functions, is it reduced susceptibility development to the biocides themselves, or is the main risk addessed whether biocides co-select for antibiotic resistance? Co-selection is currently not regulated in environmental contexts, but could from a legal perspective be considered a relevant endpoint under the EU Water Framework directive (Ågerstrand et al. 2023). However, for co-selection to occur, the concentrations of a metal or biocide need to inhibit growth of a wildtype bacterium, which makes our collected MIC data highly valuable in such regulatory efforts. One could for example envisage a somewhat simliar approach to that for assessing risks for antibiotic resistance selection based on comprehensive MIC data (Palme and Larsson 2016) given that growth inhibition is a prerequisite both for direct selection and co-selection. However, one should note the greater uncertainty with regards to co-selection as it also required shared or coupled resistance mechanisms. It is worth noting that both growth-reduction and co-selection may occur concentrations well below the MIC, pointing to other important gaps with regards to defining safe exposure concentrations for metals and biocides.

While we think the present work represents one step forward, similar efforts should be made to collect a comparable dataset focusing on MBCs. This would represent an important addition to the field, enabling more comprehensive studies on reduced susceptibility selection as well as biocide and metal efficacy. It is likely that the same limitations and gaps encountered in the MIC dataset would also be present in such an MBC dataset.

Although MICs have inherent value on their own, they should not be interpreted as the only metric to consider, but rather as one step towards centralized datasets that support comprehensive studies based on standardized methodologies. These efforts should be expanded through the development of centralized MBC datasets, as well as the integration of other types of data, such as phenotypic, genomic, and metagenomic data, to assess ecological implications or risk assessments, especially at the community level.

In conclusion, the collected MIC dataset provides a centralized source of data that will facilitate research on reduced susceptibility to biocides and metals. The collected dataset is biased towards clinical settings and particular clinically relevant species, and it also reflects the lack of standard methodologies for biocide/metal MIC determinations, a problem that has been highlighted previously. Additionally, the data facilitates the identification of particular inconsistencies in MIC results, specifically when measurements have been taken from the same strain, and highlights the difficulty of attributing these discrepancies to any single factor. The MIC dataset represents a step forward in identifiying inconsintencies and knowledge gaps, and further emphasizes the urgent need for standarization to generate comprehensive and useful data.

## Supplementary Material

fiag075_Supplemental_Files

## Data Availability

The data gathered in the present work is available as supplementary files in the present publication.
