# Supplementary material for: Bacterial sensitivity distributions for biocides and metals
Source: FEMS Microbiol Ecol. 2026 Jul 10;102(8):fiag075. doi: 10.1093/femsec/fiag075 (PMC13377640; doi:10.1093/femsec/fiag075)
Supplement: fiag075_Supplemental_Files [file fiag075_supplemental_files.zip › 5-Supplementary_files_legends.docx]

Supplementary Figure S1. Flowchart of the protocol followed for inclusion/exclusion of data.

Supplementary Figure S2. Distribution of species based on gram-type.

Supplementary Figure S3. Number of species per familiy represented in the MIC dataset.

Supplementary Figure S4. MIC distribution of the most represented metals in the dataset. Number of datapoints per species for each metal: Copper (Cu) - S. saprophyticus (423), S. aureus (182), K. pneumoniae (85), E. coli (179); Zinc (Zn) - S. saprophyticus (422), S. aureus (180), E. coli (176); Arsenic (As) - S. enterica (197), S. aureus (423); Silver (Ag) - S. aureus (210), E. coli (187).

Supplementary file S1. Boolean combinations used on literature searches in NCBI.

Supplementary File S2. MIC data for biocides collected from literature. Each sheet corresponds to a specific biocide.

Supplementary File S3. MIC data for metals collected from the literature. Each sheet corresponds to a specific metal.

Supplementary File S4. MIC data for other compounds collected from the literature. Each sheet corresponds to a specific compound.

Supplementary File S5. Statistics associated to the distribution data represented in Fig. 3, Fig.4 and Supplementary Figure S4.

Supplementary File S6. MIC distribution plots for A) biocides, B)metals and C) individual species generated from the collected MIC dataset.
