# Supplementary material for: Bacterial sensitivity distributions for biocides and metals
Source: FEMS Microbiol Ecol. 2026 Jul 10;102(8):fiag075. doi: 10.1093/femsec/fiag075 (PMC13377640; doi:10.1093/femsec/fiag075)
Supplement: fiag075_Supplemental_Files [file fiag075_supplemental_files.zip › Supplementary_file_S1.docx]

**Supplementary file 1**. Boolean combinations used on literature searches in NCBI.

Biocide + Resistance (text word) (2018 present)

Biocide + New + Resistance + Gene (Text word) (2018 present)

“Biocide resistance gene” (Text word) (2018 present)

“Metal resistance gene” (Text word) (2018 present)

New + Metal +Resistance + gene (Text word) (2018 present)

Biocides + resistance + bacteria (Text word) (2018 present)

Biocide + cell growth + resistance (Text word) (2018 present)

Biocide + bacterial growth (Text word) (2018 present)

Disinfectant + bacterial growth (Text word) (2018 present)

Biocide + disinfectant + resistance (Text word) (2018 present)

Biocide + metal + disinfectant + resistance + gene (Text word) (2018 present)

Biocide + minimum inhibitory concentration (Text word)

Biocide + minimum inhibitory concentration + growth (Text word)

Metal + minimum inhibitory concentration (Text word)

Metal + minimum inhibitory concentration + growth (Text word)

Bacteria + disinfectant +MIC (Text word)

Bacteria + antiseptic + MIC (Text word)

Bacteria + biocide + MIC (Text word)

Bacteria + metal + MIC (Text word)

Bacteria + disinfectant + minimum inhibitory concentration (Text word)

Bacteria + antiseptic + minimum inhibitory concentration (Text word)

Bacteria + biocide + minimum inhibitory concentration (Text word)

Bacteria + metal + minimum inhibitory concentration (Text word)
