# Supplementary material for: Bacterial sensitivity distributions for biocides and metals
Source: FEMS Microbiol Ecol. 2026 Jul 10;102(8):fiag075. doi: 10.1093/femsec/fiag075 (PMC13377640; doi:10.1093/femsec/fiag075)

Supplementary File S6. MIC distribution plots for A) biocides, b)metals and C) individual species generated from the collected MIC dataset.

1. Biocides


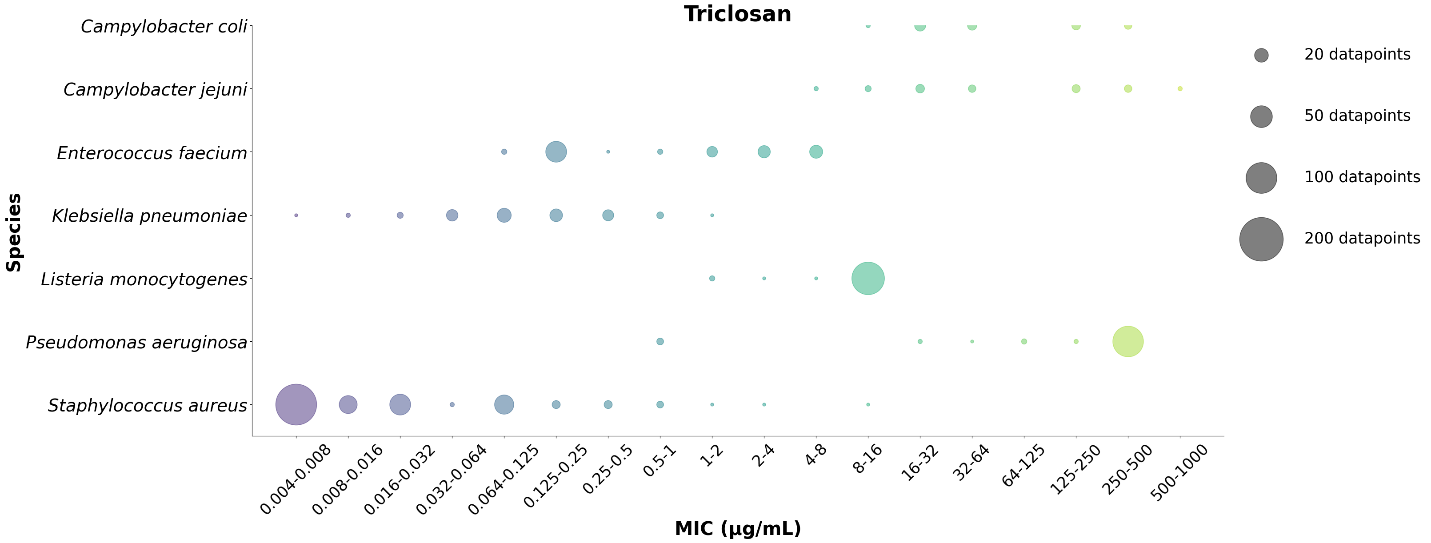


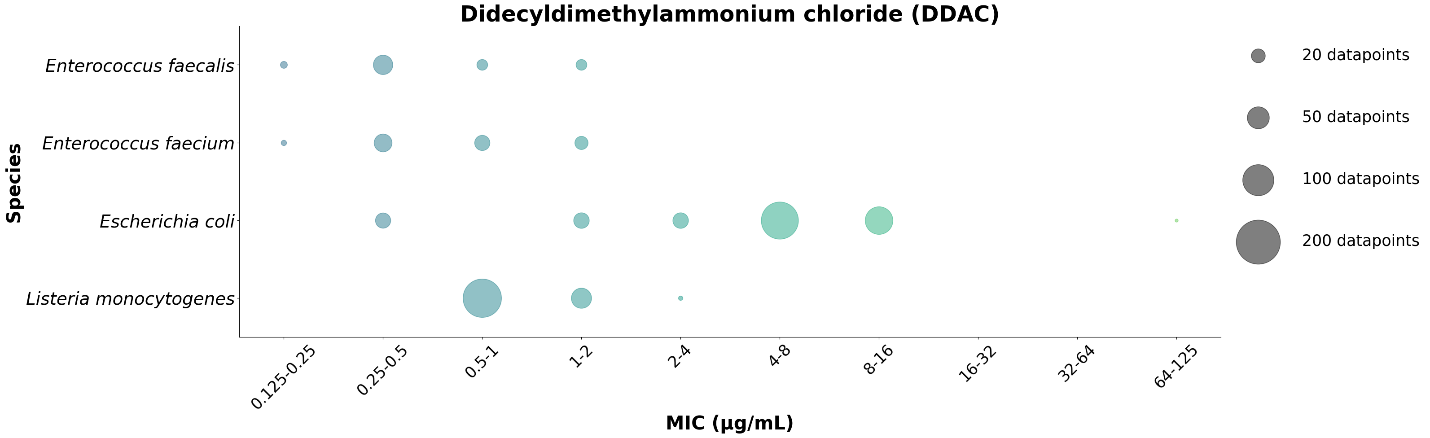


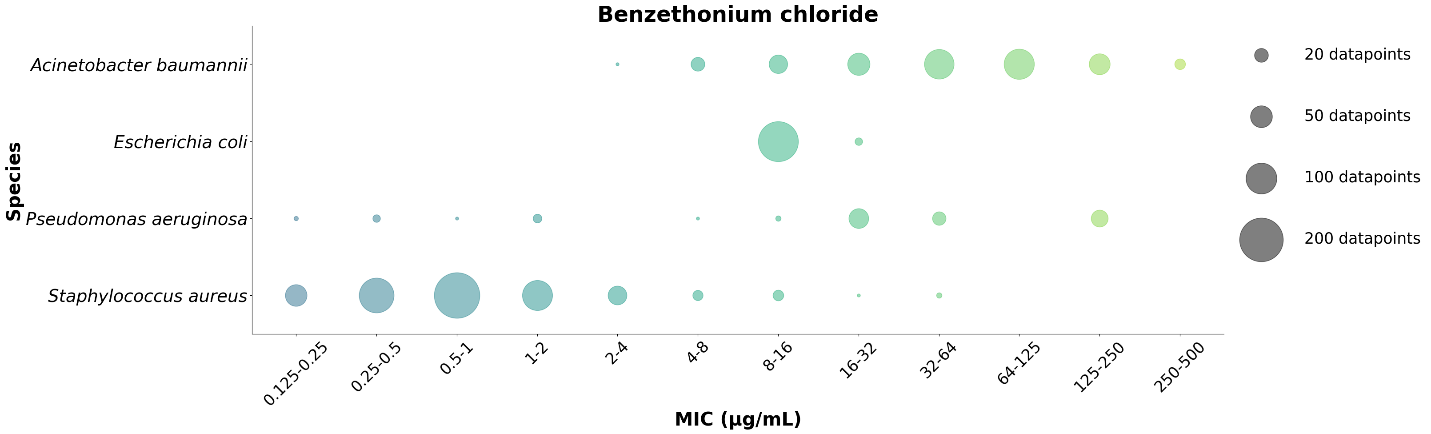


1. Metals


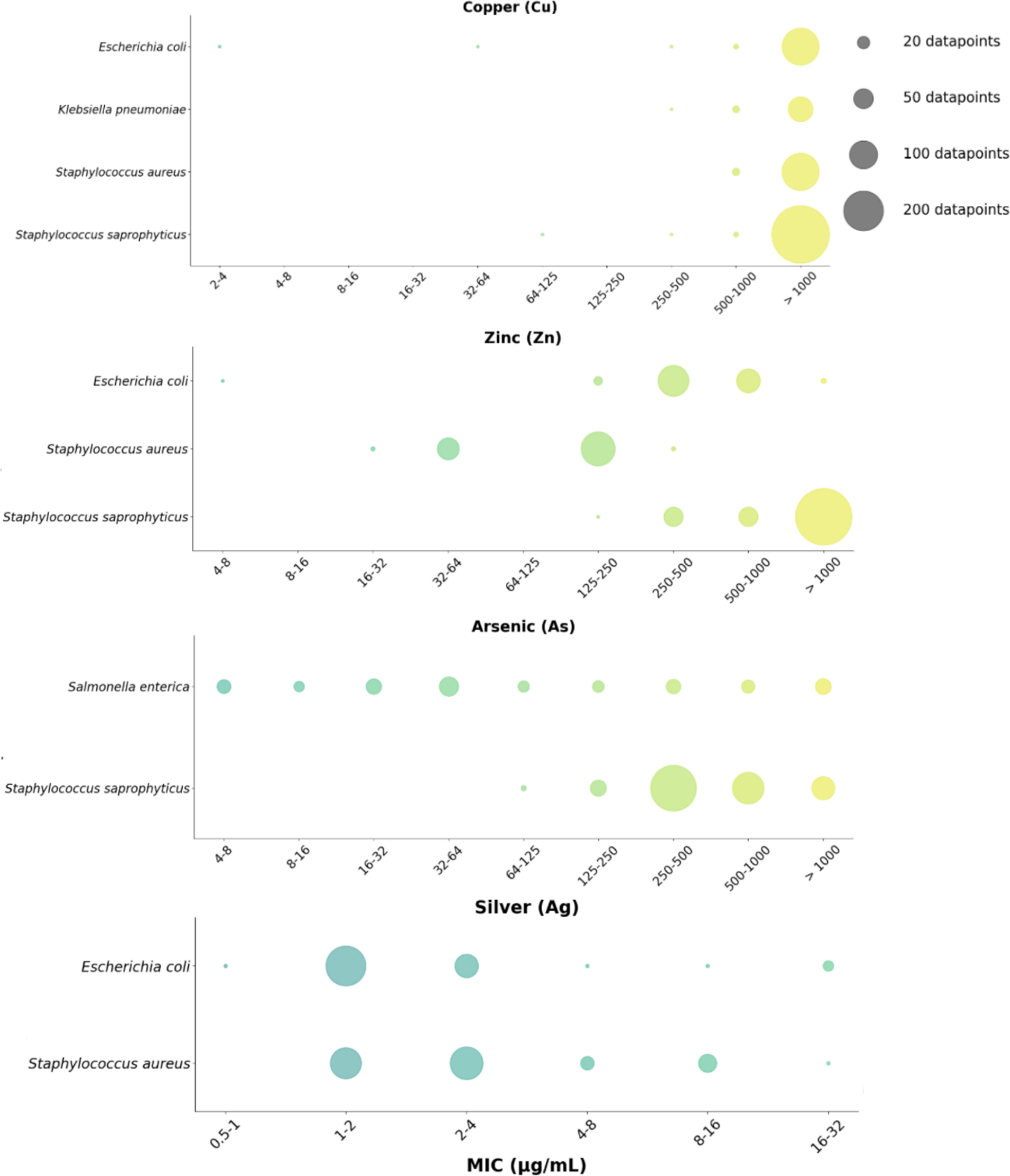


1. Individual species.


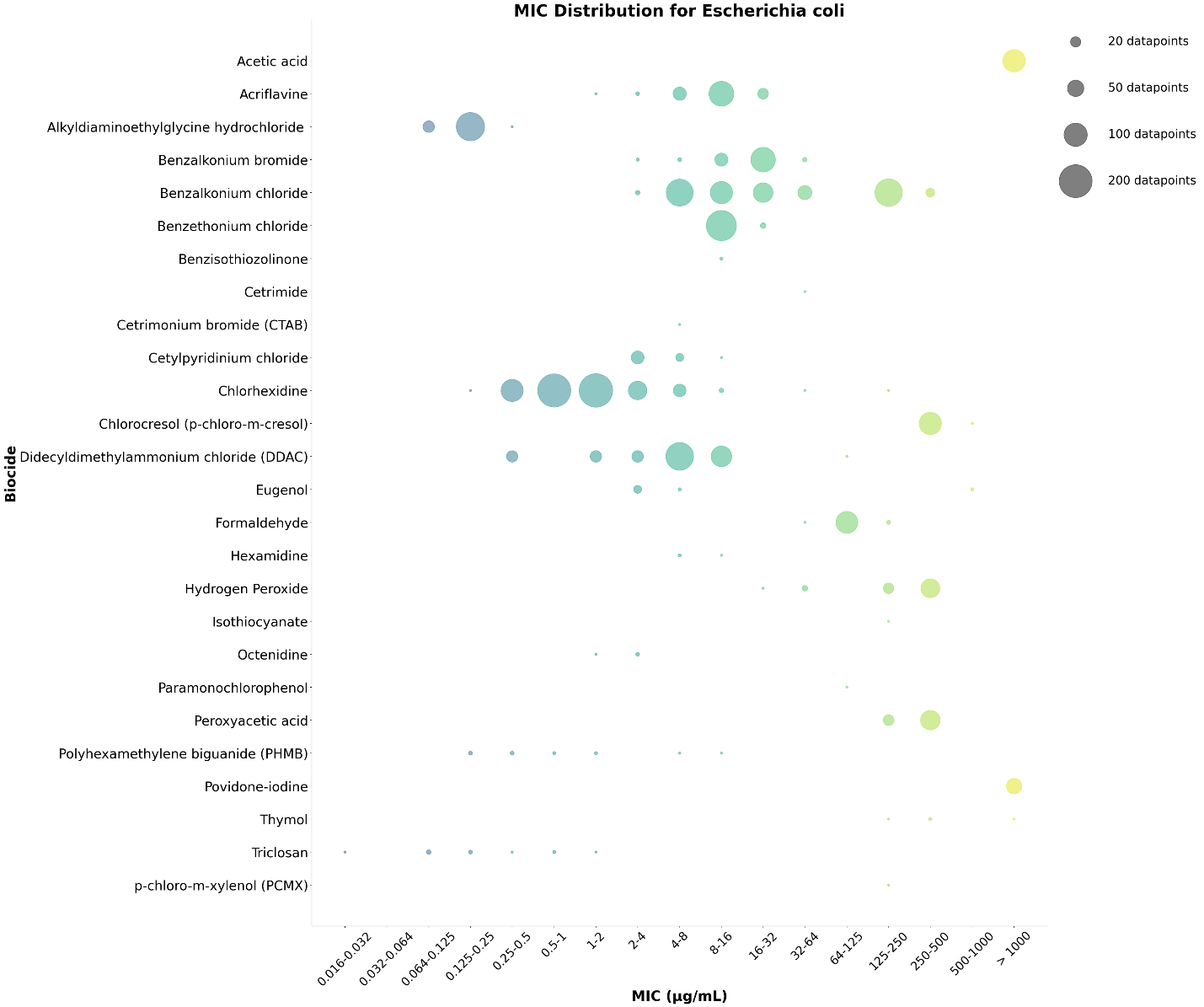


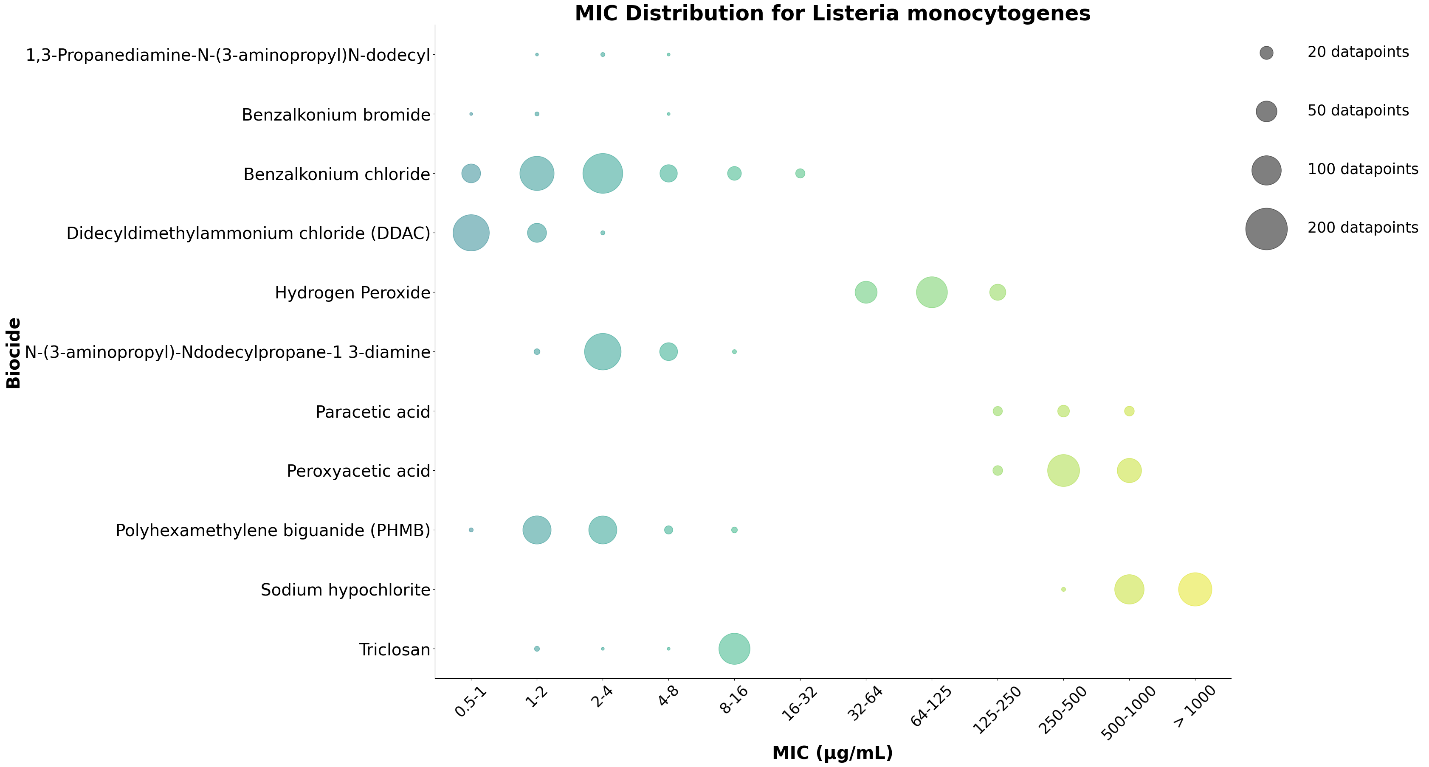


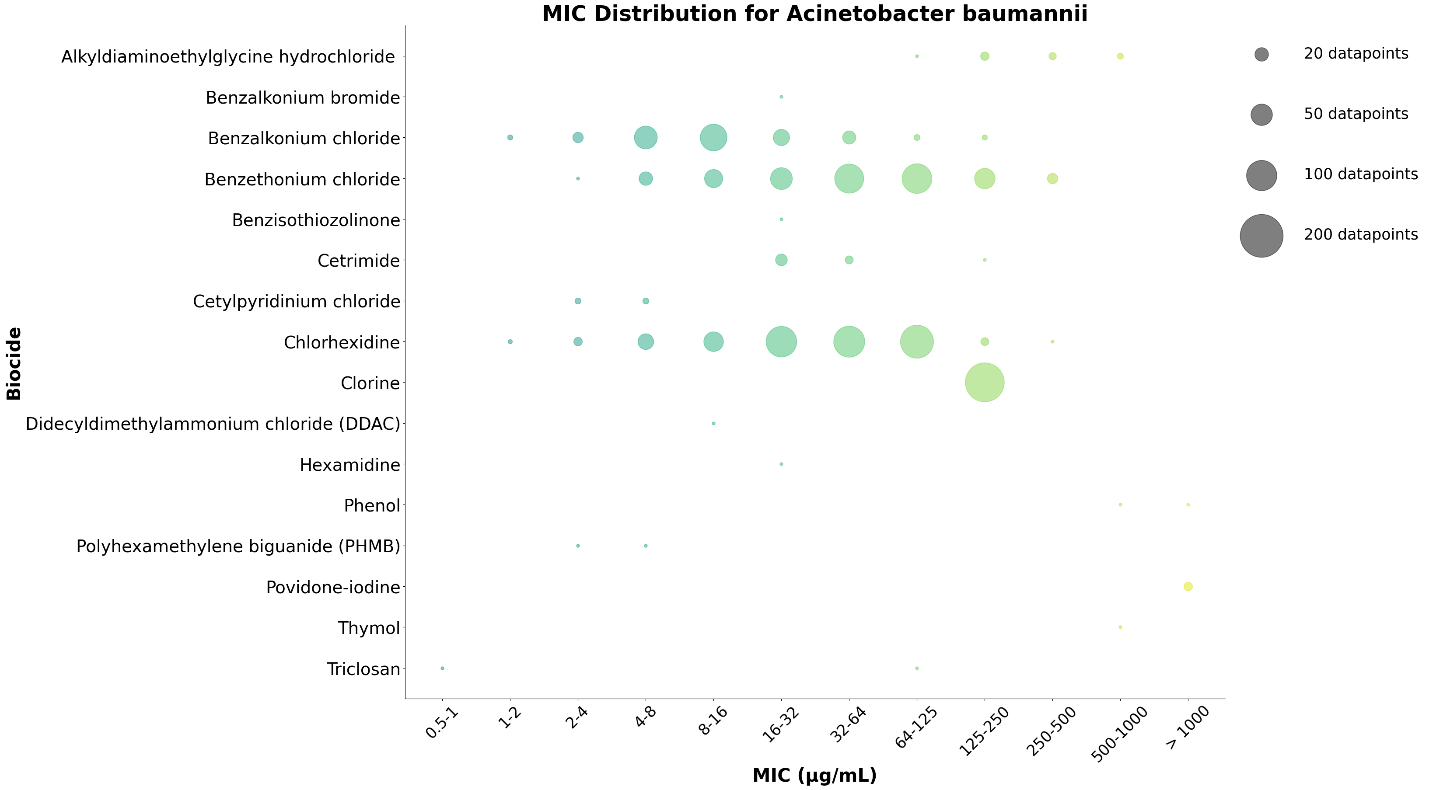


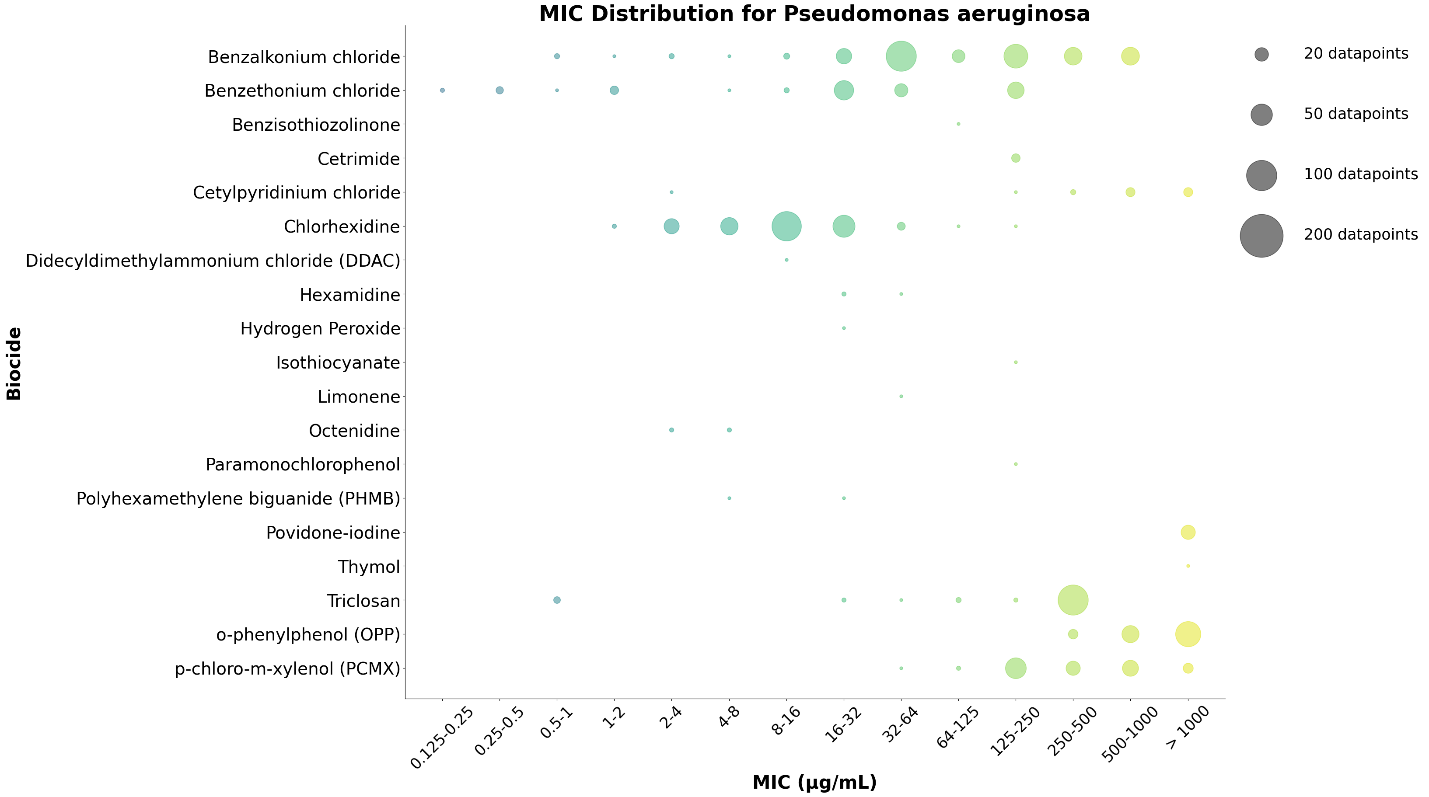

Supplement: fiag075_Supplemental_Files [file fiag075_supplemental_files.zip › Supplementary_file_S6.docx]
