# Supplementary figures and images for: Bacterial sensitivity distributions for biocides and metals
Source: FEMS Microbiol Ecol. 2026 Jul 10;102(8):fiag075. doi: 10.1093/femsec/fiag075 (PMC13377640; doi:10.1093/femsec/fiag075)

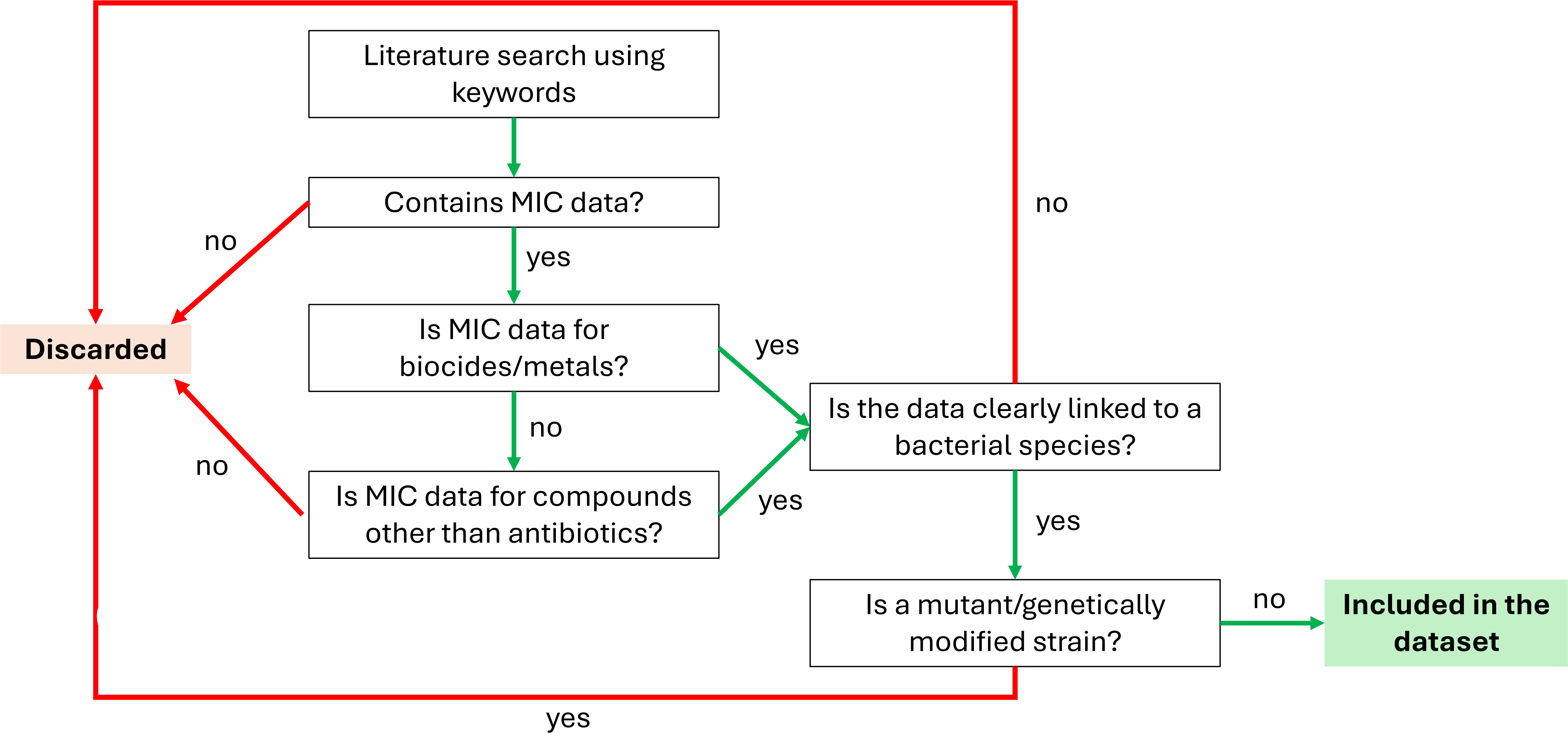

Supplement: fiag075_Supplemental_Files [file fiag075_supplemental_files.zip › FigS1.tiff]

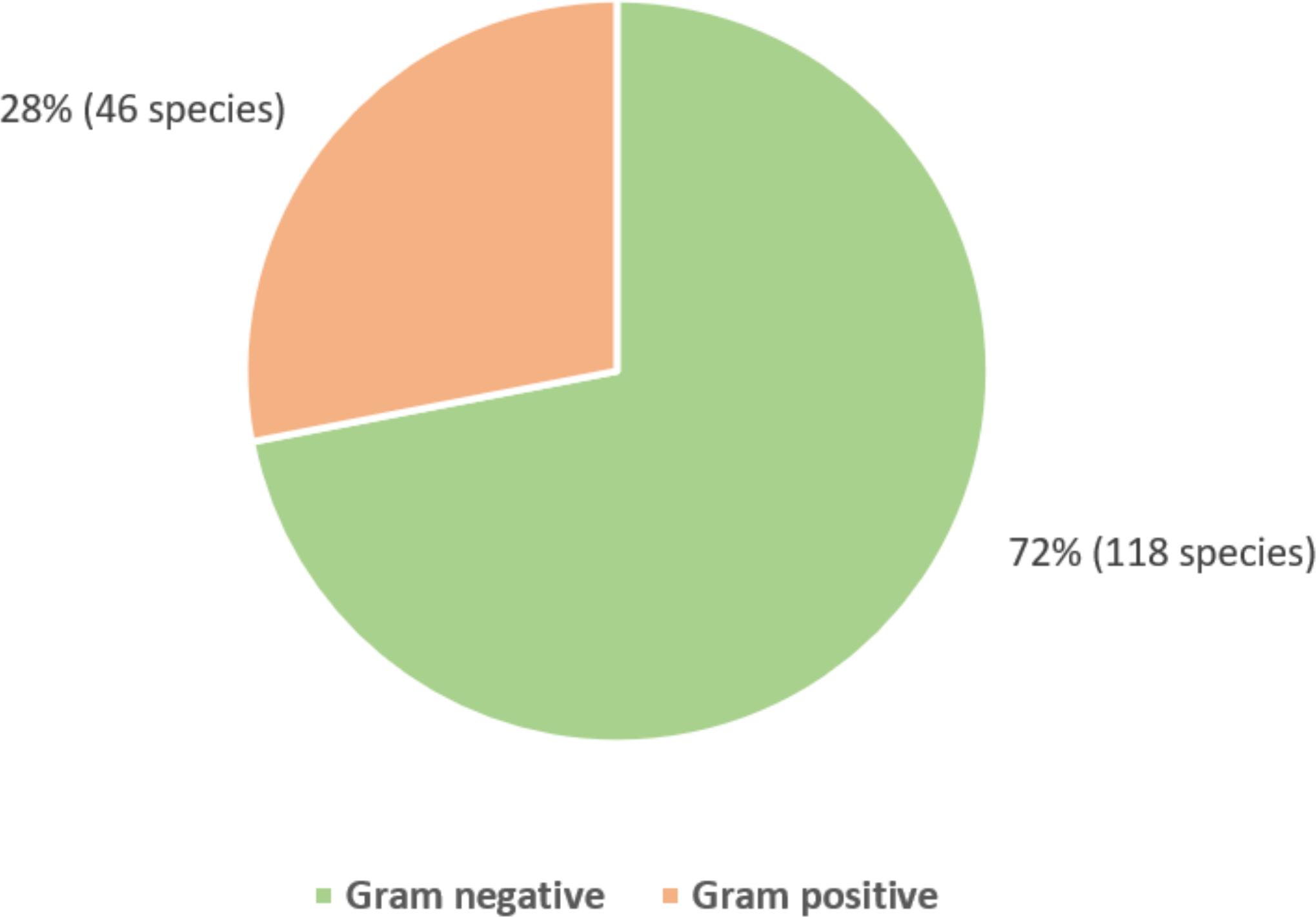

Supplement: fiag075_Supplemental_Files [file fiag075_supplemental_files.zip › FigS2.tiff]

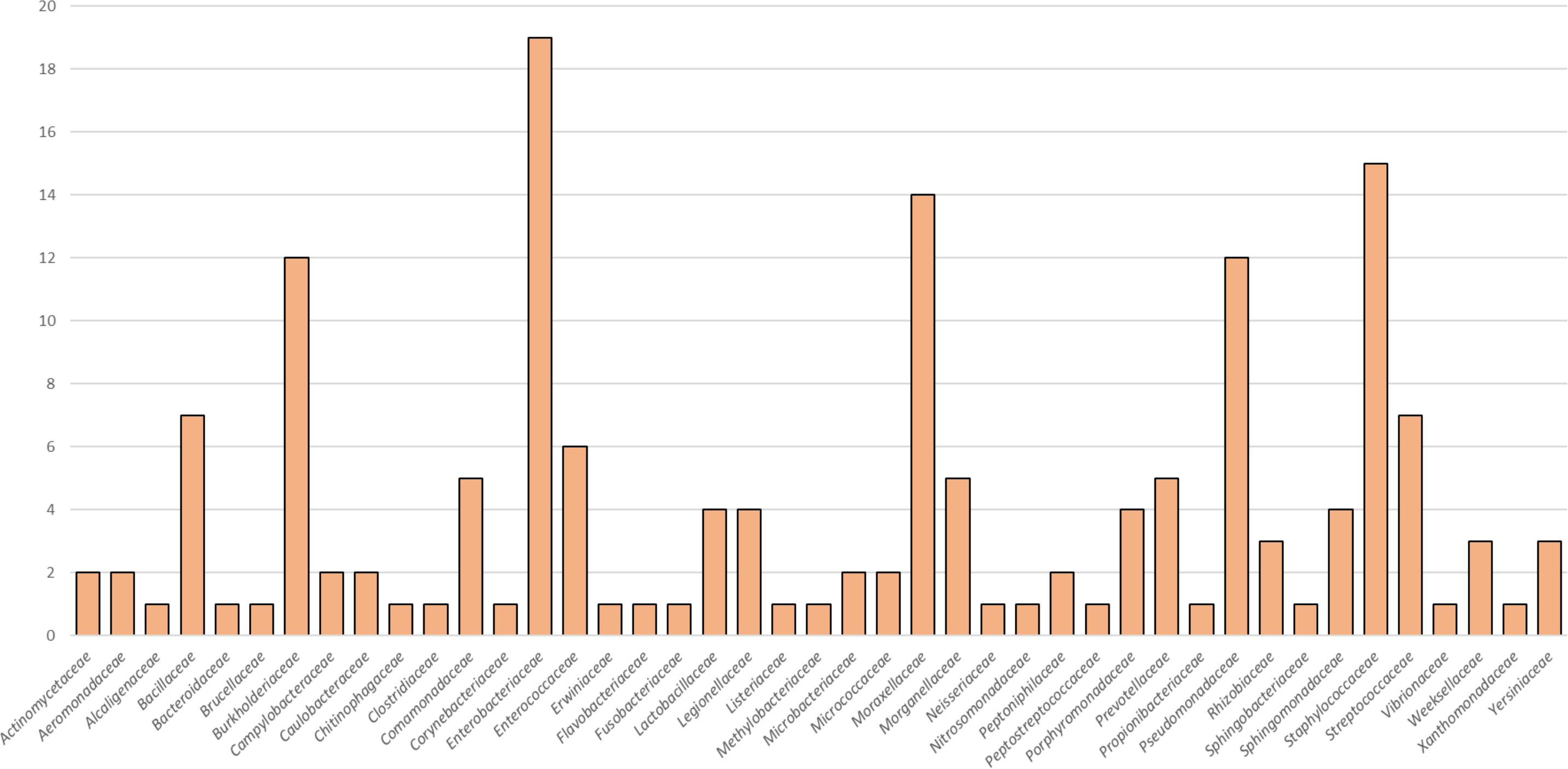

Supplement: fiag075_Supplemental_Files [file fiag075_supplemental_files.zip › FigS3.tiff]

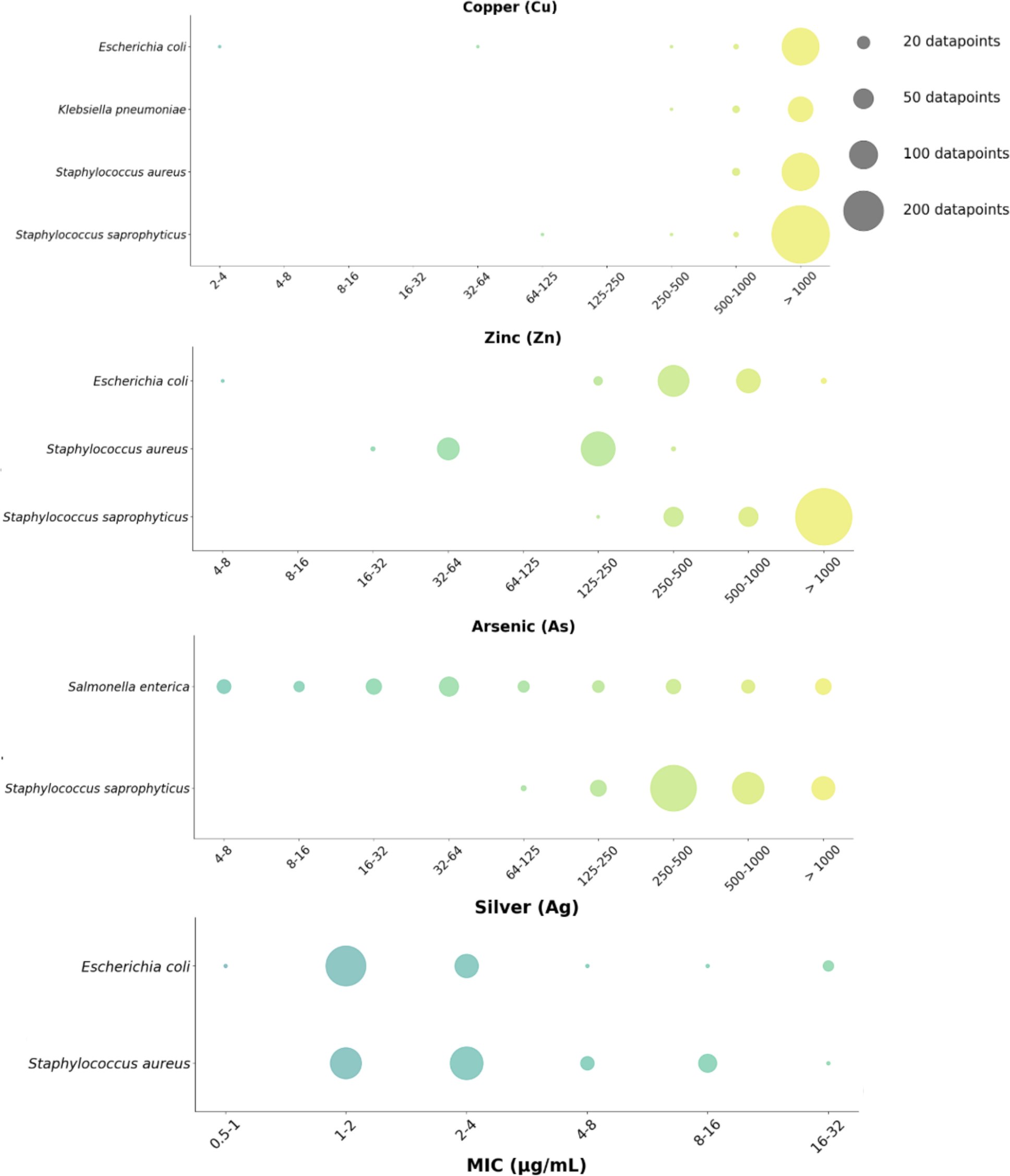

Supplement: fiag075_Supplemental_Files [file fiag075_supplemental_files.zip › FigS4.tiff]
